# Predictors for spontaneous pleurodesis in patients with indwelling pleural catheters for malignant pleural effusion: a safety net hospital experience

**DOI:** 10.1177/17534666251318844

**Published:** 2025-02-17

**Authors:** Saad Farooq, Sabiha Armin, Jordan E. Killingsworth, Akriti Agrawal, Adishwar Rao, Rosa M. Estrada-Y-Martin, Sujith V. Cherian

**Affiliations:** Division of Critical Care, Pulmonary and Sleep Medicine, Department of Internal Medicine, McGovern Medical School, The University of Texas at Houston, Houston, TX, USA; Department of Internal Medicine, McGovern Medical School, The University of Texas at Houston, Houston, TX, USA; Department of Internal Medicine, McGovern Medical School, The University of Texas at Houston, Houston, TX, USA; Department of Internal Medicine, Robert Packer Hospital/Guthrie Clinic, Sayre, PA, USA; Department of Internal Medicine, Robert Packer Hospital/Guthrie Clinic, Sayre, PA, USA; Division of Critical Care, Pulmonary and Sleep Medicine, Department of Internal Medicine, McGovern Medical School, The University of Texas at Houston, Houston, TX, USA; Division of Critical Care, Pulmonary and Sleep Medicine, Department of Internal Medicine, McGovern Medical School, The University of Texas at Houston, Houston, TX 77030, USA

**Keywords:** indwelling pleural catheter, malignant, medical thoracoscopy, pleural effusion

## Abstract

**Background:**

Malignant pleural effusion (MPE) affects approximately 150,000 patients in the United States each year and usually signifies advanced-stage cancer. The optimal treatment remains a challenge but indwelling pleural catheters (IPC) offer several advantages and may help achieve spontaneous pleurodesis (SP) in some patients.

**Objectives:**

We aim to investigate the predictors of SP among patients with MPE, particularly in a resource-limited community-based safety net hospital.

**Design:**

This is a retrospective cohort study done at a community-based safety net hospital.

**Methods:**

Adults diagnosed with or suspected of having MPE between January 2015 and December 2023 who underwent IPC placement were included. Data was collected retrospectively from December 2023 to June 2024. Data encompassed demographics, imaging, post-procedural complications, pleural fluid analysis, oncology treatment history, and utilization of medical thoracoscopy without chemical pleurodesis (MTWCP) for diagnosis.

**Results:**

A total of 173 patients underwent IPC insertion. Most of our patients were women (64.2%), and Latin American (65.9%), with a mean age of 55.3 years. The most common type of primary cancer was breast (28.9%) followed by lung (23.1%) and lymphoma (6.9%). Pleural fluid characteristics such as glucose, eosinophils, Lactate Dehydrogenase (LDH), and protein concentration were not significantly associated with SP. Most patients had low Eastern Cooperative Oncology Group scores of 0–2 (64.6%) and low LENT (Lactate Dehydrogenase (L), Eastern Cooperative Oncology Group (E) Performance Score, Neutrophil-to-Lymphocyte Ratio (N), and Tumor type (T) score) scores of 0–4 (59%). Lower scores (better functional status) were significantly associated with SP. Post-IPC chemotherapy and/or radiotherapy and immunotherapy were significantly associated with SP, adjusted odds ratio (OR) 7.295 (95% CI: 3.05–17.4, *p* = 0.001) and adjusted OR 6.261 (95% CI: 2.73–14.36, *p* = 0.001) respectively. MTWCP was also a predictor of SP with an adjusted OR of 4.031 (95% CI: 1.452–11.19, *p* = 0.007).

**Conclusion:**

Our study is the first to assess predictors of SP in a resource-limited safety net hospital representing under-represented and underserved patients. We identify several factors associated with higher rates of SP such as higher functional status, MTWCP, chemotherapy, immunotherapy, and radiation post-IPC placement. The study findings can help clinicians consider IPC placement and guide them regarding the duration and possible complications of IPC. MTWCP appears to improve the success of SP. Further studies are needed to assess these findings further.

## Plain language summary

Factors associated with increased rates of lung and chest wall fusion in patients who had an indwelling pleural catheter inserted for a malignant pleural effusion

• The lungs and chest wall are lined with thin membranes called pleura. The area between the lung and chest wall, i.e- the pleural space contains a small amount of fluid, approximately 1 teaspoon. Pleural effusion is an abnormal build-up of fluid within this space.

• Malignant pleural effusions (MPE) defined as the spread of cancer cells within the pleural space may develop in 15% of patients with cancer.

• MPE may cause symptoms such as shortness of breath, cough, and chest pain.

• Indwelling pleural catheters are small, flexible tubes made from silicone that can be inserted into the chest cavity and allow drainage of MPE even while at home.

• These indwelling pleural catheters may cause spontaneous pleurodesis (SP) which occurs when the two layers of the lining of the lung fuse together.

• We studied various factors that may be associated with a faster onset of spontaneous pleurodesis.

• We found that functional status, chemotherapy, immunotherapy and radiation post-IPC placement and placement of IPC after medical thoracoscopy (a procedure where a camera inserted into the pleural space for visualization and biopsies after fluid drainage) were associated with significantly higher rates of spontaneous pleurodesis.

## Introduction

Around 150,000 patients are diagnosed with malignant pleural effusions (MPEs) each year in the United States which accounts for an estimated financial cost of 5 billion dollars per year.^1,2^ MPE develops in 15% of all patients with cancer; breast and lung cancer account for the majority of cases.^3^ These effusions usually signify advanced-stage cancers, thus management of MPE is often aimed at improving quality of life and reducing symptoms such as dyspnea, cough, and chest pain.^3^ Therapeutic interventions for MPE include recurrent thoracentesis, chemical or mechanical pleurodesis, or insertion of indwelling pleural catheters (IPC) with patient preference and functional status often driving the suggested intervention.^1^ Pleurodesis refers to the merging of the parietal and visceral pleura, which eliminates the pleural space and does not allow further accumulation of pleural fluid and thus may help reduce symptoms such as dyspnea.^1^ IPCs are soft, flexible tubes made from silicone, subcutaneously inserted, and tunneled to the chest cavity that allow outpatient drainage of recurrent MPE and help manage symptoms such as shortness of breath without frequent thoracentesis. These catheters are small, mobile, and safe to drain at home allowing patients to continue their daily activities and avoid recurrent hospitalizations. This makes them a popular choice to manage MPE.^4^ While preventing MPE with pleurodesis using chemicals such as talc remains a viable option, IPCs are increasingly being used as first-line therapy.^5^ Both these interventions are supported by professional society guidelines for the management of dyspnea in patients with MPE. IPCs often result in decreased length of hospital stay and have reduced risk of treatment failure.^2^ Over time, IPCs can cause spontaneous pleurodesis (SP) and once this occurs it may be removed.

The predictors of SP among patients with MPE are poorly defined. The pathways and mechanisms involved in how SP occurs remain unknown, but it is hypothesized that pleurodesis occurs by inducing inflammation in the pleural space. Moreover, other factors that likely promote pleurodesis include the development of coagulation and fibrogenesis. The frequency of SP in patients with IPCs varies considerably in reported literature ranging anywhere from 27% to 70%.^4,5^ Although this wide range could be from study-specific interventions and protocols, it also signifies the considerable heterogeneity among patients, different types and stages of cancers, and an interplay of poorly understood factors such as Eastern Cooperative Oncology Group (ECOG) performance scale, pleural fluid biochemistries, drainage frequency of pleural fluid, the use of combined talc and IPC, chemotherapy/immunotherapy/ radiation.^6^ Addala et al. investigated SP rates among 119 patients out of which 45% achieved SP. The authors found that pleural fluid protein and patients undergoing concurrent chemotherapy or immunotherapy had a statistically significant increase in the rate of SP.^6^ An interesting study was done by Psallidas et al. as part of the TIME-2 trial. The authors investigated novel proteins in the pleural fluid that could increase the rate of SP. They found 97 proteins, which were significantly different between the group that achieved SP versus the group that did not achieve SP.^7^

Of note, most of the prior studies investigating SP in patients with IPCs have been performed among patients in high-volume tertiary care centers with adequate follow-up and economic resources. Some of the interventions mentioned above such as daily drainage are associated with high economic costs, thus limiting its applicability in settings with lower economic resources.

We sought to investigate predictors of SP among patients with MPE post-IPC placement in a low-resource setting catering to a large indigent and economically disadvantaged population. A comprehensive understanding of these factors enables clinicians to incorporate them into medical decision-making while considering the issues regarding the patient populations they serve.

## Materials and methods

**Figure 1.**
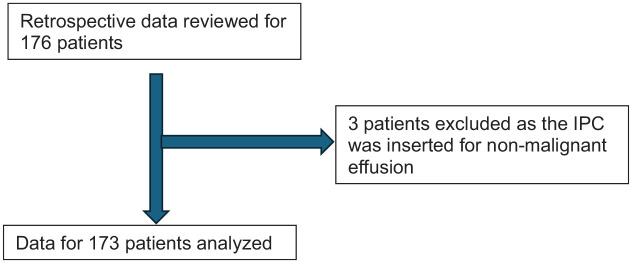
Flow chart depicting patient selection for study.

We conducted a retrospective cohort study of IPCs from a community-based safety net hospital (Lyndon B. Johnson Hospital). The reporting of this study conforms to the Strengthening the Reporting of Observational Studies in Epidemiology (STROBE) statement^8^ (Supplemental Material). A safety net hospital is a hospital, often located in poor and underserved communities which primarily provides care to uninsured or underinsured patients along with patients with low income. Most of our patients are from a clinically under-represented and underserved population in Houston, Texas. We used ICD-10 codes for MPE and a prospectively maintained procedural logbook to identify cases with IPC placement. We included adult patients aged 18 years or older, diagnosed with or suspected (pleural effusion in the setting of known malignancy) of having MPE between January 2015 and December 2023, who underwent IPC placement. We excluded patients who had IPC inserted for other etiologies such as palliation in nonmalignant effusion. One hundred seventy-six patients were identified out of which three were excluded as they had IPC inserted for nonmalignant pleura effusion (Figure 1).

Since this was a retrospective cohort study, a formal sample size was not calculated, and the relatively smaller sample size may affect the statistical significance of the results.

### Procedures

**Figure 2.**
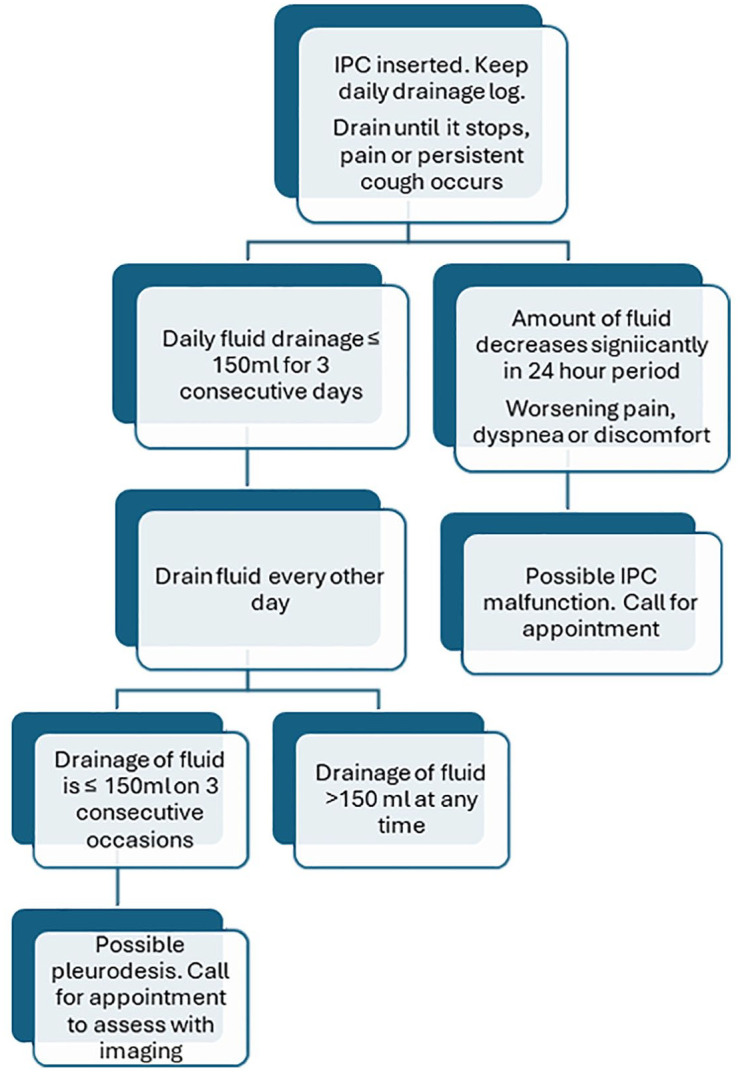
Flowchart depicting institutional drainage of indwelling pleural catheter.

All patients had IPCs (Pleurx X; CareFusion, Becton, Dickinson and Company, Franklin Lakes, NJ, USA) placed under local anesthesia, or after medical thoracoscopy which was performed under moderate sedation. Procedures were performed as either inpatient or outpatient, under sterile conditions without any concurrent administration of antibiotics. After the insertion of IPC, the patient and at least one family member were educated regarding the care of the catheter, and they were discharged only after education had occurred. The drainage protocol for the patients included daily drainage, once fluid drainage was <150 mL, then the patients were instructed to space out the drainage to every other day, followed by every 3–4 days. Once fluid drainage was <150 cc on three consecutive attempts within 2 weeks, the patient was instructed to call the pulmonary department and schedule an appointment for evaluation either with a chest X-ray or thoracic ultrasound. If minimal pleural fluid was seen by bedside thoracic ultrasound, then the IPC was removed. Although we assessed other factors such as the presence of loculations and lung sliding on ultrasound, these did not affect our decision to remove the IPC. For non-draining catheters, and concomitant imaging showing the presence of fluid, an attempt for drainage with intrapleural tissue plasminogen activator (tPA) is performed (Figure 2).

If the IPC continued draining, the patients continued follow-up in the pulmonary clinic every 2 months where we would ensure that the site looked clean and dry and that the patients had been receiving the IPC supplies. At each visit, patients are encouraged to drain the IPC as per our drainage regimen and we discussed any issues that we could address related to the IPC drainage. If we removed the catheter, the patients could stop routine follow-up after 6 months in the pulmonary clinic.

### Data collection

Data collected included demographics, imaging parameters, post-procedural complications, pleural fluid analysis metrics, oncology treatment history, and the utilization of medical thoracoscopy without chemical pleurodesis (MTWCP) for the diagnosis of cancer.

### Definitions

Pleural effusions are categorized as malignant if malignant cells are detected on the cytology of pleural fluid or biopsy during medical thoracoscopy. Initially, IPCs were placed during the early part of this period in patients with a high pretest probability of MPE; cytology negative, but lymphocyte predominant exudate in the presence of known malignancy), since medical thoracoscopy was introduced in our institution later in 2019.

IPC-related SP was defined as minimal or no output through a patent IPC, associated with a minimal or no effusion on chest imaging with either X-ray or ultrasound.

IPC-related pleural space infections were defined as any of the following:

1. Obvious pus drained from the catheter.
2. Presence of clinical symptoms consistent with infection with positive pleural fluid gram stain or cultures.
3. Presence of clinical symptoms consistent with infection along with pleural fluid biochemical characteristics supportive of infection such as elevated lactate dehydrogenase, low glucose, or low pH.

All pleural fluid samples sent for biochemical and microbiologic studies were obtained through the IPC itself. The following post-IPC outcomes were studied: Infection, re-expansion pulmonary edema, pneumothorax, IPC dislodgement, tPA use, death before IPC removal, loss to follow-up, removal due to infection, IPC removal.

### Statistical analysis

Demographic variables included age, gender, and ethnicity, as well as other characteristics such as the ECOG score, LENT score, duration of IPC, pleural fluid LDH, pleural fluid protein, pleural fluid glucose, pleural fluid eosinophils, repeat IPC, primary cancer type, effusion side, post-IPC infection, trapped lung/pneumothorax ex vacuo, pleural nodule, medical thoracoscopy, chemotherapy pre-IPC, chemoradiotherapy (chemotherapy and/or radiotherapy) post-IPC, and immunotherapy post-IPC. ECOG and LENT scores were categorized into low-risk (0–2 and 0–4) and high-risk (3–4 and 5–7) groups, respectively.

The data was analyzed using the Pearson Chi-square test to compare categorical variables between the two groups and the two-sample *t*-test for continuous variables. Age, LENT score, and various pleural fluid parameters were categorized into groups for this statistical analysis. Furthermore, a secondary analysis after excluding all patients who died in less than 30 days was also performed.

Cox models and logistic regression models were utilized to compute the unadjusted and adjusted odds ratios (ORs). A competing risk analysis and Kaplan–Meier curves to estimate the time to pleurodesis were performed. Lastly, a sensitivity analysis was performed to ensure the robustness of the results. Stata 18 BE (Stata Corp LLC, College Station, TX, USA) was used for statistical analysis, with statistical significance set at an alpha of 0.05. The models were tested for multicollinearity by calculating the variance inflation factor (VIF) and for goodness of fit by calculating the Akaike Information Criterion.

## Results

A total of 173 IPCs were placed from January 2015 to December 2023. Most patients were women (64.2%), and Latin American (65.9%), with a mean age of 55.3 years. Most patients had low ECOG scores of 0–2 (64.6%), and low LENT scores of 0–4 (59%), signifying better functional status were associated with SP (Table 1). Given the retrospective nature, some variables had missing values such as pleural fluid LDH and pleural fluid glucose, as fluid was only sent for cytology in these cases, but these were <5% of the cohort.

**Table 1.** Baseline demographics and descriptive data for patients who underwent IPC insertion.

Demographic	No SP	SP	Total (*n*, %)	*p*-Value
Total, *n* (%)	119 (68.8)	54 (31.2)	173 (100)	**n/a**
Age in years (mean, 95% CI)±SD	54.9 (52.4–57.5) ± 13.9	56.0 (53.1–58.9) ± 10.7	55.3 (53.3–57.2) ± 13.0	0.615
Gender, *n* (%)				
Female	74 (42.8)	37 (21.4)	111 (64.2)	0.421
Male	45 (26.0)	17 (9.8)	62 (35.8)	
Ethnicity, *n* (%)				
Hispanic	79 (45.7)	35 (20.2)	114 (65.9)	0.840
Non-Hispanic	40 (23.1)	19 (11.0)	59 (34.1)	
Pleural fluid LDH				
<200	32 (23.0)	12 (8.6)	44 (31.7)	0.607
⩾200	65 (46.8)	30 (21.6)	95 (68.3)	
Pleural fluid LDH (mean ± SD)	834 ± 1461	637 ± 951	774 ± 1328	0.426
Pleural fluid protein, *n* (%)				
<3.5	22 (12.7)	5 (2.9)	27 (15.6)	0.121
⩾3.5	97 (56.1)	49 (28.3)	146 (84.4)	
Pleural fluid protein (mean ± SD)	4.13 ± 1.23	4.50 ± 0.78	4.24 ± 1.12	0.077
Pleural fluid glucose, *n* (%)				
<60	24 (13.9)	8 (4.6)	32 (18.5)	0.401
⩾60	95 (54.9)	46 (26.6)	141 (81.5)	
Pleural fluid glucose (mean ± SD)	86.1 ± 45.2	89.7 ± 43.5	87.2 ± 44.6	0.668
Pleural fluid eosinophils, *n* (%)				
<5%	91 (52.6)	40 (23.1)	131 (75.7)	0.733
⩾5%	28 (16.2)	14 (8.1)	42 (24.3)	
Pleural fluid eosinophils (mean ± SD)	0.821 ± 2.87	1.07 ± 2.46	0.899 ± 2.74	0.622
ECOG categories, *n* (%)				
Low (0–2)	62 (37.8)	44 (26.8)	106 (64.6)	**0.002**
High (3–4)	48 (29.3)	10 (6.1)	58 (35.4)	
LENT categories, *n* (%)				
Low (0–4)	63 (36.4)	39 (22.5)	102 (59.0)	**0.017**
High (5–7)	56 (32.4)	15 (8.7)	71 (41.0)	
Cancer type, *n* (%)				
Breast	35 (20.2)	15 (8.7)	50 (28.9)	0.213
Lymphoma	5 (2.9)	7 (4.0)	12 (6.9)	
Lung	29 (16.8)	11 (6.4)	40 (23.1)	
Others	50 (28.9)	21 (12.1)	71 (41.0)	
Effusion side, *n* (%)				
Right	38 (22.0)	19 (11.0)	57 (32.9)	0.302
Left	34 (19.7)	20 (11.6)	54 (31.2)	
Bilateral	47 (27.2)	15 (8.7)	62 (35.8)	
Duration of IPC in days (median, range)	15 (1–923)	68 (3–553)	110 (1–922)	0.600
Duration of IPC (>30 days), *n* (%)				
Yes	4 (6.1)	47 (71.2)	51 (77.3)	<**0.001**
No	9 (13.6)	6 (9.1)	15 (22.7)	
Trapped lung/Pneumothorax ex vacuo, *n* (%)				
Yes	21 (12.1)	4 (2.3)	25 (14.5)	0.076
No	98 (56.6)	50 (28.9)	148 (85.5)	
Chemotherapy pre-IPC, *n* (%)				0.598
Yes	82 (48.2)	35 (20.6)	117 (8.8)	
No	35 (20.6)	18 (10.6)	53 (31.2)	
Chemoradiotherapy post-IPC, *n* (%)				<**0.001**
Yes	50 (26.7)	46 (29.1)	96 (55.8)	
No	68 (39.5)	8 (4.7)	76 (44.2)	
Immunotherapy post-IPC, *n* (%)				<**0.001**
Yes	40 (24.8)	40 (24.8)	80 (49.7)	
No	71 (44.1)	10 (6.2)	83 (50.3)	
Medical thoracoscopy, *n* (%)				
Yes	13 (7.6)	13 (7.6)	26 (15.1)	**0.027**
No	105 (61.0)	41 (23.8)	146 (84.9)	

ECOG, Eastern Cooperative Oncology Group; IPC, indwelling pleural catheters; SP, spontaneous pleurodesis. The bold values are values which are statistically significant with *P* < 0.05.

The most common type of primary cancer was breast (50, 28.9%) followed by lung (40, 23.1%) and lymphoma (12, 6.9%). Pleural effusions were mostly unilateral (64.1%) with almost similar numbers on the right and left side. Pleural fluid analysis revealed that a majority had LDH ⩾200 U/L (95, 68.3%) and most had protein ⩾3.5 g/dL (146, 84.4%) reflecting exudative effusions. Pleural fluid glucose was mostly ⩾60 mg/dL (141, 81.5%), and eosinophils in the pleural fluid were mostly <5% (131, 75.7%). Fourteen patients (8.1%) developed a pleural space infection after IPC placement whereas four patients (2.3%) had re-expansion pulmonary edema. Table 2 shows some of the other post-IPC placement outcomes. Table 3 outlines different causative organisms for IPC-related pleural space infections. Staphylococci accounted for more than half of these infections.

**Table 2.** Outcome data for patients post-IPC insertion.

Outcomes	No SP, *n* (%)	SP, *n* (%)	Total, *n* (%)	*p*-Value
Infection				
Yes	11 (6.4)	3 (1.7)	14 (8.1)	0.410
No	108 (62.4)	51 (29.5)	159 (91.9)	
Re-expansion pulmonary edema				
Yes	2 (1.2)	2 (1.2)	4 (2.3)	0.423
No	115 (67.3)	50 (29.4)	167 (97.7)	
Pneumothorax				
Yes	8 (4.7)	3 (1.8)	11 (6.5)	0.773
No	109 (64.1)	52 (29.4)	159 (93.5)	
IPC dislodgement				
Yes	3 (1.8)	2 (1.2)	5 (2.9)	0.688
No	113 (66.5)	52 (30.6)	165 (97.1)	
tPA use				
Yes	14 (8.2)	9 (5.3)	23 (13.5)	0.402
No	103 (60.2)	45 (26.3)	148 (86.5)	
Death before removal				
Yes	75 (43.4)	0 (0)	75 (43.4)	<**0.001**
No	44 (25.4)	54 (31.2)	98 (56.6)	
Lost to follow-up				
Yes	25 (14.5)	0 (0)	25 (14.5)	< **0.001**
No	94 (54.3)	54 (31.2)	148 (85.5)	
IPC removed				<0.001
Yes	7 (4.0)	54 (31.2)	**61 (35.3)**	
No	112 (64.7)	0 (0.0)	**112 (64.7)**	

IPC, indwelling pleural catheters; SP, spontaneous pleurodesis; tPA, tissue plasminogen activator. The bold values are values which are statistically significant.

**Table 3.** Etiology of infections.

Micro-organisms	Number of patients
Methicillin-sensitive *Staphylococcus aureus*	4
Methicillin-resistant *Staphylococcus aureus*	1
*Staphylococcus epidermidis*	2
*Staphylococcus ludgunensis*	1
*Streptococcus constellatus*	1
Gamma hemolytic streptococcus	1
Micrococcus	1
Acinetobacter	1
Proteus	1
Bacillus	1

Repeat IPC placement was required in only two (1.2%) patients. Pleural nodule(s) were present in almost half of the patients (83, 48.3%). The median duration of IPC among patients who achieved SP was 68 days (ranging from 1 to 922 days). A small number of patients developed trapped lungs or pneumothorax ex vacuo (25, 14.5%). Most patients received chemotherapy pre-IPC placement (117, 68.8%).

**Figure 3.**
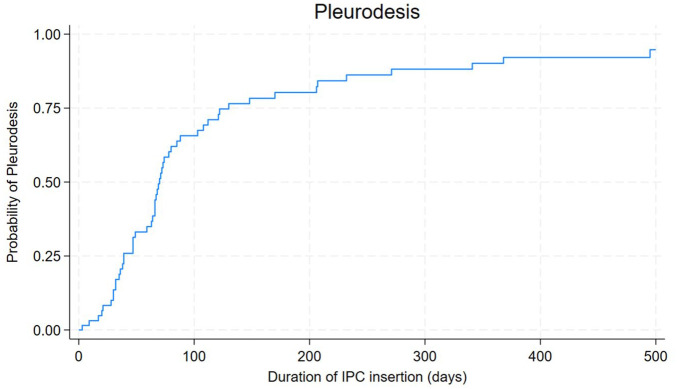
Kaplan–Meier curve showing overall time to pleurodesis in patients with indwelling pleural catheters.

SP was achieved in 54 cases (31.2%). The rates of SP did not significantly differ across types of malignancies. Pleural fluid LDH was not significantly different among those with SP and those without, nor were pleural fluid protein, glucose, and other biochemical markers. Figure 3 highlights the Kaplan–Meier curve for time to SP.

Most patients who developed SP had received chemotherapy and/or radiotherapy post-IPC (46, 29.1%) whereas only eight patients (4.7%) who did not receive chemoradiotherapy achieved SP. Similarly, most patients who developed SP had received immunotherapy (40, 24.8%), whereas only 10 patients (6.2%) who did not receive immunotherapy achieved SP. Medical thoracoscopy was performed on 26 patients. SP was achieved in 13 (7.6%) of those patients versus 13 (7.6%) who did not. The adjusted OR was 4.031 (95% CI: 1.45–11.2), *p* = 0.007 (Table 3).

Multivariable regression analysis and Cox models showed that the unadjusted OR of SP among those who received chemoradiotherapy post-IPC was 7.820 (95% CI: 3.393–18.02), *p* < 0.001, compared to those who did not and did not change after adjusting for age, gender, ethnicity, LENT categories, chemotherapy pre-IPC and medical thoracoscopy, the adjusted OR was 7.295 (95% CI: 3.053–17.43), *p* = 0.001. The unadjusted OR of SP among those who received immunotherapy post-IPC was 7.100 (95% CI: 3.210–15.706), *p* < 0.001, compared to those who did not and did not change after adjusting for age, gender, ethnicity, LENT categories, chemotherapy pre-IPC and medical thoracoscopy, the adjusted OR was 6.261 (95% CI: 2.731–14.36), *p* < 0.001 (Tables 4 and 5). Table 6 shows that these factors stayed congruent even when excluding patients with IPCs who died in less than 30 days after placement.

**Table 4.** Unadjusted and adjusted ORs using multivariable logistic regression for various predictors for the development of SP.

Predictors of pleurodesis	Development of SP		Sensitivity analysis: Alternative models
	Unadjusted OR (95% CI), *p*-value	Adjusted^a^ OR (95% CI), *p*-value	Adjusted^a^ OR (95% CI), *p*-value
Medical thoracoscopy	2.561 (1.095–5.987), ***p* = 0.030**	4.031^b^ (1.452–11.187), ***p* = 0.007**	2.978 (1.185–7.48),^c^ ***p* = 0.02**
Chemotherapy pre-IPC	0.830 (0.415–1.659), *p* = 0.598	0.698^d^ (0.331–1.471), *p* = 0.344	0.790 (0.384–1.63),^c^ *p* = 0.522
Chemoradiotherapy post-IPC	7.820 (3.393–18.021), ***p* < 0.001**	7.295^a^ (3.053–17.43), ***p* ⩽ 0.001**	8.069 (3.468–18.77),^c^ ***p* ⩽ 0.001**
Immunotherapy post-IPC	7.100 (3.210–15.706), ***p* < 0.001**	6.261^a^ (2.731–14.36), ***p* ⩽ 0.001**	7.24 (3.245–16.16),^c^ ***p* ⩽ 0.001**
LENT categories	0.433 (0.216–0.868), ***p* = 0.018**	0.386^e^ (0.187–0.798), ***p* = 0.010**	0.434 (0.215–0.875),^c^ ***p* = 0.02**
ECOG categories	0.294 (0.134–0.642), ***p* = 0.002**	0.273^e^ (0.121–0.617), ***p* = 0.002**	0.302 (0.137–0.663),^c^ ***p* = 0.003**

Sensitivity analysis with more variables included in last column.

The adjusted model includes the covariates:

a Age, gender, ethnicity, LENT categories, chemotherapy pre-IPC, medical thoracoscopy.

b Age, gender, ethnicity, LENT categories, chemotherapy pre-IPC.

c Age, gender, ethnicity.

d Age, gender, ethnicity, LENT categories.

e Age, gender, ethnicity, chemotherapy pre-IPC.

The models were unlikely to have multicollinearity since the VIF was <10 for all. AIC was >6.5 for all representing good model fits.

AIC, Akaike Information Criterion; IPC, indwelling pleural catheters; SP, spontaneous pleurodesis; VIF, variance inflation factor. All bold values are statistically significant.

**Table 5.** Cox model hazard ratios.

Predictors of pleurodesis	HR with 95% CI	Log-rank *p*-value	Interpretation
Medical thoracoscopy	2.04 (1.16–3.57), *p* = 0.013	0.011	Medical thoracoscopy has higher chances of removal of IPC
Chemotherapy before IPC	1.04 (0.62–1.75), *p* = 0.089	0.89	No difference
Chemo-radio therapy after IPC	2.87 (1.65–4.97), *p* < 0.01	<0.001	Chemoradiotherapy has higher chance of removal of IPC
Immunotherapy after IPC	3.27 (1.89–5.66), *p* < 0.001	<0.001	Immunotherapy has higher chance of removal of IPC
LENT score high versus low/moderate	0.48 (0.23–1.02), *p* = 0.057	0.052	No difference
ECOG 3, 4 versus 0, 1, 2	0.39 (0.21–0.72), *p* = 0.002	0.0017	ECOG 0, 1, 2 has higher chance of removal of IPC

ECOG, Eastern Cooperative Oncology Group; HR, hazards ratio; IPC, indwelling pleural catheters.

**Table 6.** Data analysis with exclusion of patients who died within 30 days.

Duration >30 day	Development of SP	
	Unadjusted OR (95% CI), *p*-value	Adjusted^a^ OR (95% CI), *p*-value
Medical thoracoscopy	2.649 (1.058–6.628), ***p* = 0.037**	3.936^b^ (1.326–11.68), ***p* = 0.014**
Chemotherapy pre-IPC	0.776 (0.377–1.599), *p* = 0.492	0.671^c^ (0.310–1.452), *p* = 0.311
Chemoradiotherapy post-IPC	7.726 (3.184–18.75), ***p* < 0.001**	7.316^a^ (2.908–18.41), ***p*** **=** **<0.001**
Immunotherapy post-IPC	6.937 (3.007–16.00), ***p* < 0.001**	6.231^a^ (2.597–14.95), ***p*** **=** **<0.001**
LENT categories	0.459 (0.222–0.949), ***p* = 0.036**	0.411^d^ (0.192–0.877), ***p* = 0.021**
ECOG categories	0.216 (0.0887–0.528), ***p* < 0.001**	0.190^d^ (0.0739–0.491), ***p* = <0.001**

The adjusted model includes the covariates:

a Age, gender, ethnicity, LENT categories, chemotherapy pre-IPC, medical thoracoscopy.

b Age, gender, ethnicity, LENT categories, chemotherapy pre-IPC.

c Age, gender, ethnicity, LENT categories.

d Age, gender, ethnicity, chemotherapy pre-IPC.

The models were unlikely to have multicollinearity since the VIF was <10 for all. AIC was >6.5 for all representing good model fits.

ECOG, Eastern Cooperative Oncology Group; IPC, indwelling pleural catheters; SP, spontaneous pleurodesis; VIF, variance inflation factor. All statistically significant values are made in bold.

## Discussion

SP was achieved in 32% of cases. Our study identified several predictors of SP. Chemoradiotherapy and immunotherapy post-IPC placement were associated with the development of SP among patients with MPE whereas chemotherapy pre-IPC was not. Although previous studies have clearly shown increased survival among patients who receive chemotherapy before and after IPC insertion, the impact on achieving SP is less clear.^9^ Mitchell et al. found no significant difference in time to removal of IPC for those receiving chemotherapy versus those who did not; however, their study only included patients with breast cancer, associated with higher rates of SP.^10^ In this study, immunotherapy was also associated with an increased chance of pleurodesis.^10^ Wang et al. reported that Epidermal Growth Factor Receptor (EGFR)-targeted therapy in patients with non-small cell lung cancer was associated with earlier removal of IPC although, they did not find increased rates of SP in the cohort that received immunotherapy which they postulated was due to a relatively slower onset of immunotherapy compared to EGFR-targeted therapies and possible differential inflammatory response induced by both drugs.^11^

Practically, the IPC is removed when SP is achieved, hence we extrapolate that the duration of IPC insertion is approximately indicative of the time to SP development. In our study, the median duration of IPC among those who developed SP was 68 days (ranging from 1 to 922 days). A range of different time durations for the development of SP have been reported in the literature.^9,12–14^ Our institution, which serves as a safety net hospital with limited resources, employs an initial daily drainage regimen followed by an alternate-day drainage regimen, once the output is <150 cc, which may explain the longer time to achieve pleurodesis in our cohort. Furthermore, since we are a safety net hospital, the time to start chemo and immunotherapy is often longer compared to other centers which also can prolong the time to achieve SP. Wahidi et al. reported higher rates of SP at 12 weeks in the group that drained the pleural effusion daily compared to the group that did alternative-day drainage (47% vs 24%, *p* = 0.03). The time to SP was also shorter in the daily drainage arm compared to the alternate-day drainage arm (54 days vs 90 days, *p* = 0.005).^14^ Although this study showed a clear advantage of daily drainage in achieving SP, this may not be practically feasible in resource-limited settings such as ours due to economic considerations. Furthermore, in the TIME-2 trial, the rate of SP in patients with IPC was 51%; however, the mean IPC drainage frequency during the first 42 days was only twice weekly which shows relatively high SP rates may still be achieved without daily drainage.^15^ Daily drainage is, however, still recommended by professional guidelines when IPC removal and achieving SP is a primary goal.^16^ Rates of SP though initially reported to be higher in the range of 45%–60%, are now believed to be lower in the range of 25%–30% (IPC Plus and AMPLE studies) which was seen in our cohort as well.^17,18^

Although LENT scores have been utilized in prior studies to predict survival in patients with MPE, the data on its effect on SP is less clear.^19^ In our study, LENT categories, though not achieving statistical significance were associated with increased odds of achieving SP. In multiple studies, ECOG of 0–2 was significantly associated with increased rates of IPC removal, thus IPC removal is more likely in patients with good functional status, which was highlighted in our study as well.^11,20,21^ This is probably because patients with better functional status have improved survival and can likely receive more intensive treatment thus increasing the opportunity for SP to occur.^22,23^

MTWCP, when adjusted for possible confounders, was significantly associated with SP although our sample size was limited, with only 15% of patients at our center undergoing this procedure. Notably, medical thoracoscopy was only introduced in our institution in 2019, and given the increase in critical care demands from COVID-19, true implementation of the procedure only started later in 2022. In a retrospective cohort study among patients with MPE, MTWCP in patients with ECOG of 2 or less was associated with higher SP rates (87% vs 63%, *p* = 0.001) and lesser median time to SP (38 days vs 56 days, *p* = 0.04), like our cohort.^20^ This may be secondary to the increased inflammation and bleeding associated with pleural biopsies which may facilitate pleurodesis.

The two main limitations of our study are the retrospective nature and having been conducted at a single center which limits the generalizability of our findings. Survival data was not studied after IPC removal or after the development of SP to elucidate whether the development of SP translated into improved survival (e.g., at 90 days or 1 year).

## Conclusion

Functional status (ECOG and LENT score categories), chemoradiotherapy, and immunotherapy post-IPC placement, as well as MTWCP, were associated with higher odds of SP, whereas chemotherapy pre-IPC was not. These and other predictors highlighted in the study help clinicians estimate the treatment success of IPCs in terms of the development of SP, the potential duration of IPCs, and the possible complications of IPC placement. This is the first study to assess predictors of SP in a resource-limited safety net hospital catering to an under-represented and underserved population, which is relevant in its applicability to community-based hospitals, particularly in low-income and resource-limited parts of the world. Moreover, studies involving such patients are limited- highlighting the importance of our study. Future research is needed to develop and refine predictive models to select the best candidates for IPC placement. Furthermore, prospective studies to evaluate the role of medical thoracoscopy in SP in MPE should be performed.

## Supplemental Material

sj-docx-1-tar-10.1177_17534666251318844 – Supplemental material for Predictors for spontaneous pleurodesis in patients with indwelling pleural catheters for malignant pleural effusion: a safety net hospital experience Supplemental material, sj-docx-1-tar-10.1177_17534666251318844 for Predictors for spontaneous pleurodesis in patients with indwelling pleural catheters for malignant pleural effusion: a safety net hospital experience by Saad Farooq, Sabiha Armin, Jordan E. Killingsworth, Akriti Agrawal, Adishwar Rao, Rosa M. Estrada-Y-Martin and Sujith V. Cherian in Therapeutic Advances in Respiratory Disease

## Declarations

**Ethics approval and consent to participate:** The study was approved by the Institutional Review Board of McGovern Medical School—The University of Texas and the hospital (HSC-MS-23-0838). Requirement for informed consent to participate has been waived by the relevant Ethics Committee or Institutional Review Board.

**Consent for publication:** Not applicable.

**Availability of data and materials:** The raw data supporting the conclusions of this study will be made available by the corresponding author if requested.
